# Chick Embryo Partial Ischemia Model: A New Approach to Study Ischemia *Ex Vivo*

**DOI:** 10.1371/journal.pone.0010524

**Published:** 2010-05-07

**Authors:** Syamantak Majumder, M. Ilayaraja, Himabindu Reddy Seerapu, Swaraj Sinha, Jamila H. Siamwala, Suvro Chatterjee

**Affiliations:** Vascular Biology Lab, AU-KBC Research Centre, Anna University, Chennai, India; Brigham and Women's Hospital, United States of America

## Abstract

**Background:**

Ischemia is a pathophysiological condition due to blockade in blood supply to a specific tissue thus damaging the physiological activity of the tissue. Different *in vivo* models are presently available to study ischemia in heart and other tissues. However, no *ex vivo* ischemia model has been available to date for routine ischemia research and for faster screening of anti-ischemia drugs. In the present study, we took the opportunity to develop an *ex vivo* model of partial ischemia using the vascular bed of 4^th^ day incubated chick embryo.

**Methodology/Principal Findings:**

Ischemia was created in chick embryo by ligating the right vitelline artery using sterile surgical suture. Hypoxia inducible factor- 1 alpha (HIF-1α), creatine phospho kinase-MB and reactive oxygen species in animal tissues and cells were measured to confirm ischemia in chick embryo. Additionally, ranolazine, N-acetyl cysteine and trimetazidine were administered as an anti-ischemic drug to validate the present model. Results from the present study depicted that blocking blood flow elevates HIF-1α, lipid peroxidation, peroxynitrite level in ischemic vessels while ranolazine administration partially attenuates ischemia driven HIF-1α expression. Endothelial cell incubated on ischemic blood vessels elucidated a higher level of HIF-1α expression with time while ranolazine treatment reduced HIF-1α in ischemic cells. Incubation of caprine heart strip on chick embryo ischemia model depicted an elevated creatine phospho kinase-MB activity under ischemic condition while histology of the treated heart sections evoked edema and disruption of myofibril structures.

**Conclusions/Significance:**

The present study concluded that chick embryo partial ischemia model can be used as a novel *ex vivo* model of ischemia. Therefore, the present model can be used parallel with the known *in vivo* ischemia models in understanding the mechanistic insight of ischemia development and in evaluating the activity of anti-ischemic drug.

## Introduction

Ischemia is a condition in which blockade in blood flow leads to restricted oxygen and nutrient supply to a part of the body. Cardiac ischemia is the compromised blood flow and oxygen supply to heart muscle. The aim of developing ischemic animal model is to study the basic processes or potential therapeutic interventions in ischemia associated diseases, and the extension of patho-physiological knowledge, which will lead to improve medical treatment of human ischemic stroke. Ischemic stroke has a complex pathophysiology involving the interplay of many different cells and tissues such as neurons, glia, endothelium, cardiomyocytes and the immune system. These events cannot be mimicked satisfactorily in *in vitro*. Thus a large portion of stroke research is conducted on animals. Presently two ischemic animal models are well accepted by the scientific community. Artery ligation model is created in animal heart by ligating the left coronary artery [1]. Occlusion model is another well accepted model of ischemia, which is created by permanently occluding the middle cerebral artery [2]. However, these different animal models are less feasible for routine laboratory testing of ischemia effects. Additionally, preliminary screening of any anti-ischemic drug needs to be carried out by using *in vitro* models or by using other models similar to *in vivo* conditions. In the present scenario, only few *in vitro* ischemia models such as cerebral ischemia model based on organotypic hippocampal slice cultures and tissue culture ischemia model of oxygen/glucose deprivation are documented [3], [4]. However, the models are not well conceived and mostly control the oxygen level to create hypoxia in the tissue but not specifically ischemia.

In this present study, we carried out experiments to develop a novel *ex vivo* partial ischemia model by blocking the right vitelline arteries of chick embryo. The model was validated by using three tier experimental set ups 1) ischemia in the vascular bed 2) secondary ischemia in endothelial cells (EC) and cardiomyocyte cells cultured on ischemic vascular bed and 3) secondary ischemia created in caprine cardiac tissues by placing them on ischemic vascular bed. Elevated level of reactive oxygen species (ROS) and HIF-1α followed by deteriorated cell and tissue functions validated the chick embryo partial ischemia model. Further, a strong recovery of ischemia by anti-ischemic drugs validated our novel approach of developing an *ex vivo* ischemia model.

## Results

### Partial ischemia elevated the reactive oxygen species generation in tissues

**Figure 1:**
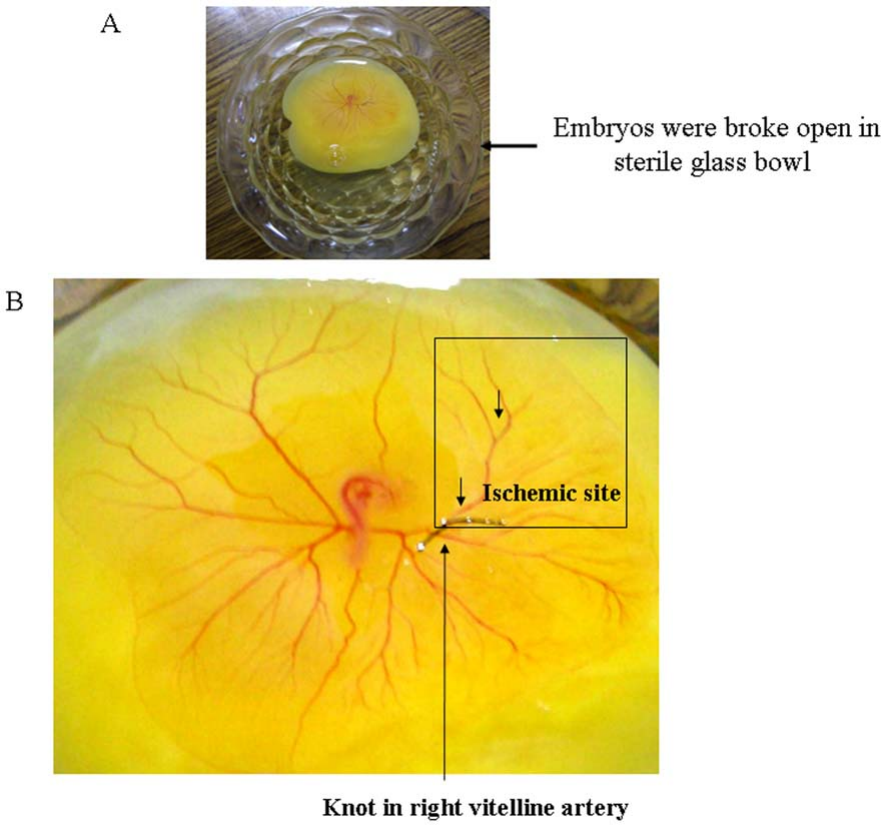
Creating partial ischemia in chick embryo vascular bed using ligation model. (A) Image showed the embryo with the whole yolk material has been placed in the sterile glass bowls. The bowls were covered with sterile glass leads and placed in a chamber maintaining 37°C and relative humidity. (B) Image represents the exact approach taken to block the blood flow to the specific area of the chick embryo vascular bed. Black arrow showed the suture that has ligated the right vitelline artery thus blocking the blood flow in the surrounding vascular bed. The box showed the ischemic area of the vascular bed.

**Figure 2:**
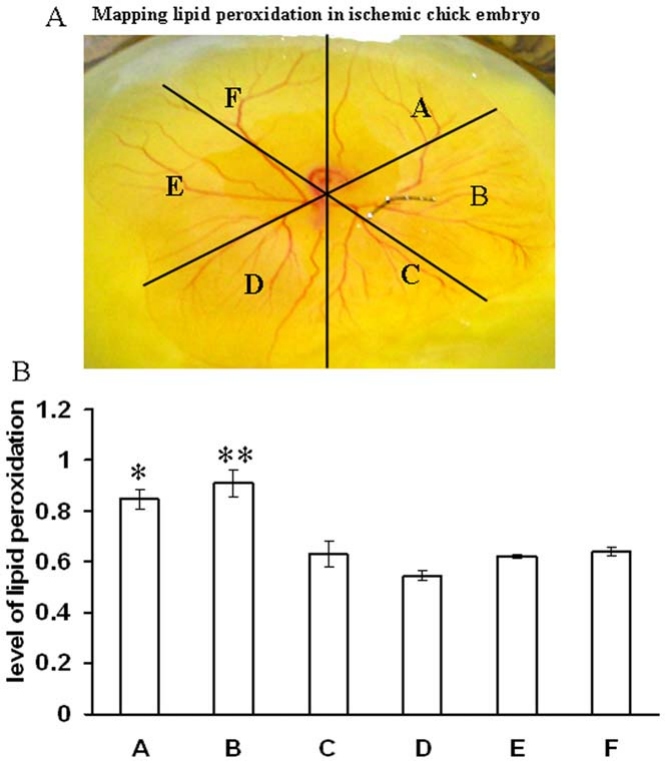
Mapping lipid peroxidation in ischemic vascular bed. (A) Representative image showed the way section has been incised out from the vascular bed of chick embryo to map the lipid peroxidation status in the ischemic vascular bed. (B) Lipid peroxidation was measured by following the TBA protocol. After ligation of right vitelline artery, embryos were incubated for 2 h. An equal six sections of the chick embryo vascular bed was taken and the lipid peroxidation was measured. Data were normalized by taking the weight of the tissues.*P<0.05 and **P<0.001 versus C,D,E and F. (n = 5).

**Figure 3:**
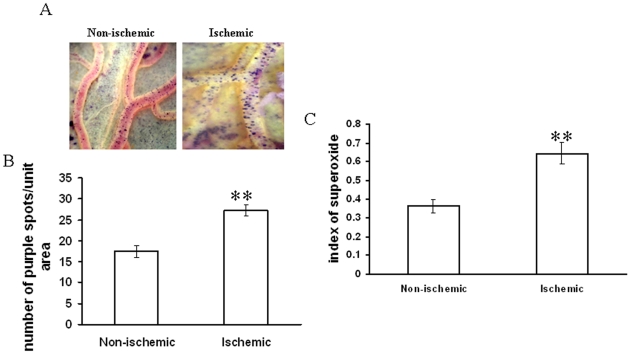
Effect of ischemia on superoxide generation in vitelline vascular tissue. (A) Vessels were probed with NBT for 1 h. Next, the images of the vessels having the purple formazen crystal were acquired with Nikon Cool Pix camera adapted to a stereo microscope. Images are the representative of 5 individual experiments. (B) The number of purple formazen crystals in the vessels was counted manually. Data were normalized by taking the area of the vessels and plotting as number of purple spots/unit area. **P<0.001 versus non-ischemic. (n = 5) (C) Ischemic vessels were incubated with NBT for 1 h followed by homogenization of the tissue and centrifugation of the suspension at 6500 g for 10 min. The supernatant was then read at 560 nm. Data were normalized with the weight of the tissue. **P<0.001 versus non-ischemic. (n = 3).

**Figure 4:**
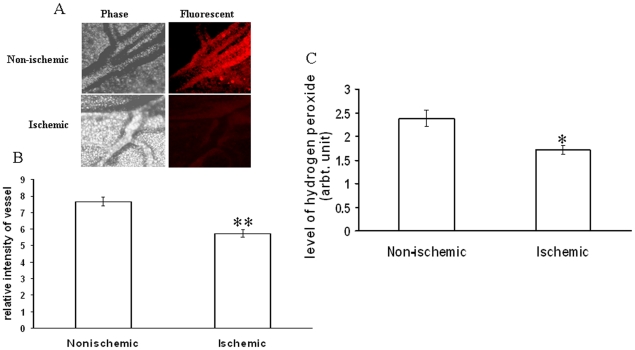
Effect of ischemia on hydrogen peroxide generation in vitelline vascular tissue. (A) Ischemia treated vessels were probed with amplex red for 30 min. Fluorescent and bright filed images were acquired with DP71 CCD camera adapted to Olympus IX71 microscope. Images are the representative of 5 individual experiments. (B) The fluorescent intensities of the images were calculated using Adobe Photoshop 7.0 and plotted. Fluorescent intensities were considered as directly proportional to the level of hydrogen peroxide produced. **P<0.001 versus non-ischemic. (n = 5) (C) Ischemic tissues were probed with amplex red for 30 min followed by homogenization and centrifugation at 6500 g for 10 min. The supernatant was then read at excitation/emission of 570/585 nm. Values obtained were normalized with the weight of the tissues. *P = 0.023 versus non-ischemic. (n = 3).

**Figure 5:**
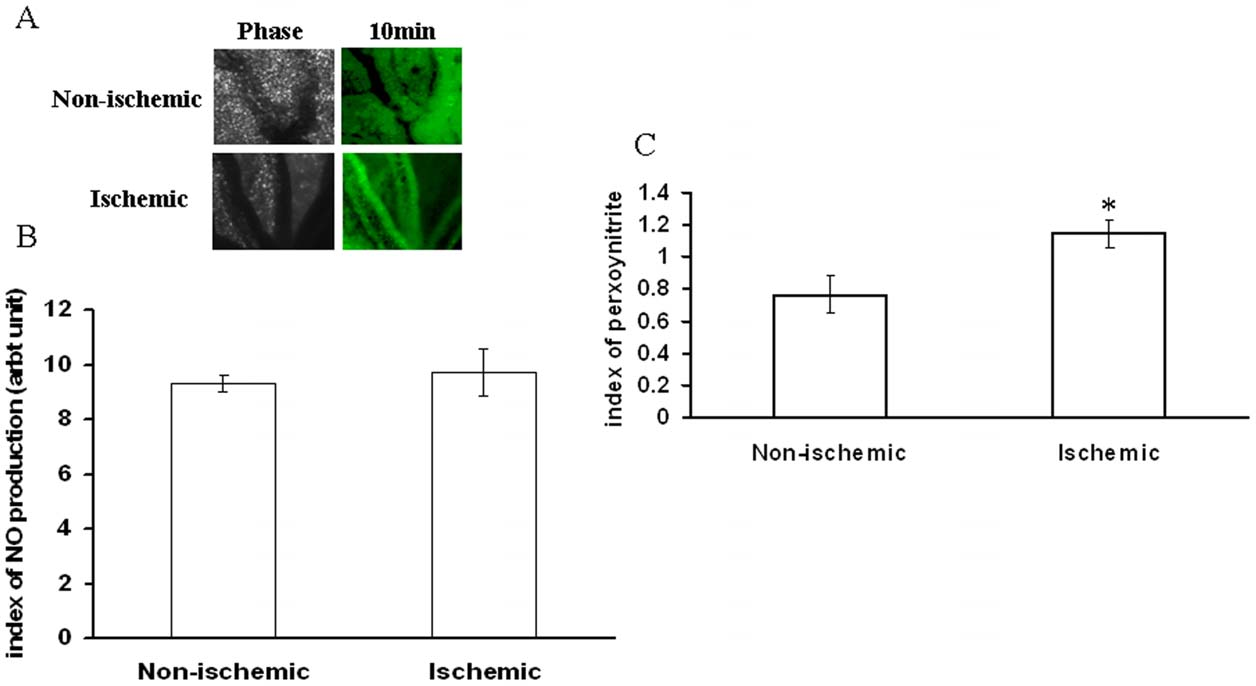
Effect of ischemia on NO generation and peroxynitrite level in vitelline vascular tissue. (A) NO production by the ischemic vessels was measured using DAF-2DA fluorescent probe. Ischemic vessels were incubated for 10 min with 1 µM DAF-2DA dissolved in HBS containing 1 mM L-arginine. Images were taken under 4X objective of the microscope. Images are the representative of 3 individual experiments. (B) The green luminosity of the fluorescent images was analyzed using Adobe Photoshop 7.0. Luminosity of the images was directly proportional to the level of NO produced thus plotted as index of NO production. **P<0.001 versus non-ischemic. (n = 3) (C) Peroxy nitrite generation was measured using DHR123 fluorescent probe. Tissues were incubated with DHR123 for 2 h followed by homogenization and centrifugation for 5 min at 6500 g. Supernatant was read at excitation/emission of 500/536 nm and the values were normalized with the weight of the tissues and plotted. *P = 0.03 versus non-ischemic. (n = 5).

**Figure 6:**
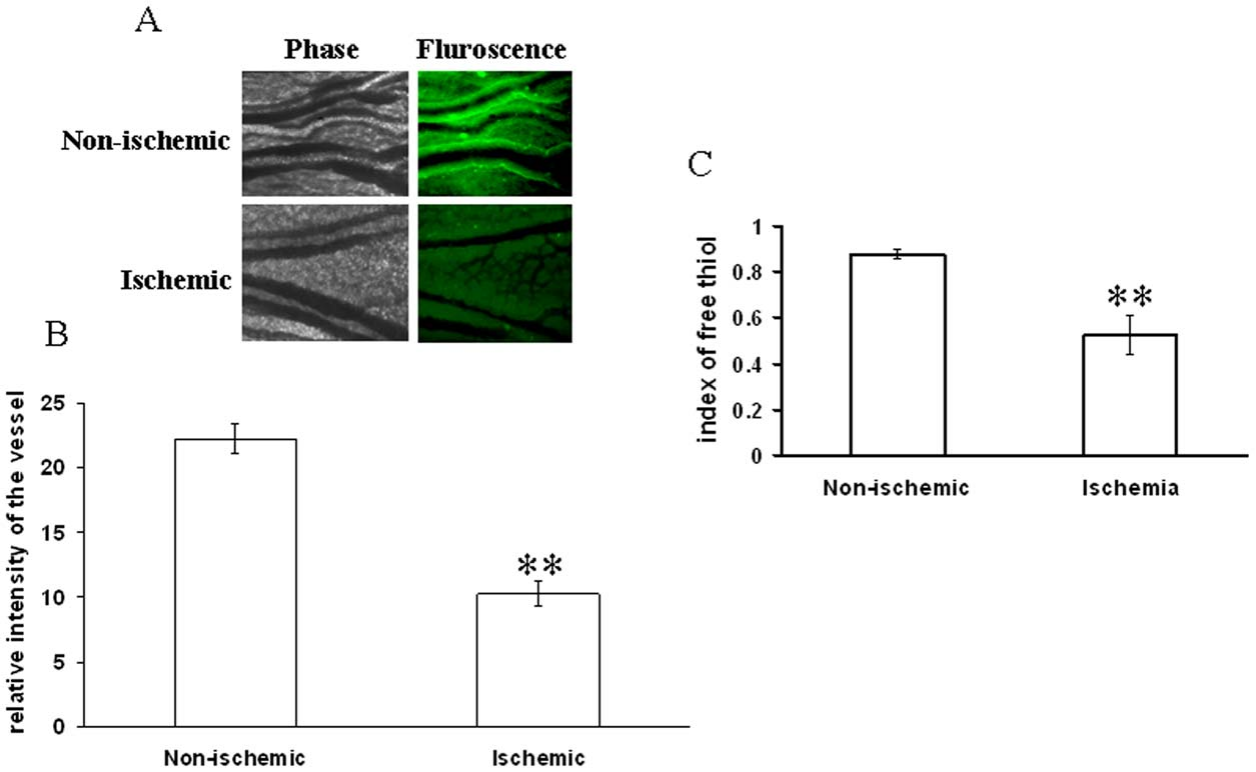
Effect of ischemia on GSH level in vitelline vascular tissue. (A) Level of GSH was measured using CMFDA fluorescent probe. The ischemic vessels were incubated with CMFDA probe for 30 min. Next, the images were acquired with DP71 CCD camera. Images are the representative of 5individual experiments. (B) The fluorescent intensities of the images were calculated using Adobe Photoshop 7.0 and plotted. Fluorescent intensities were considered as directly proportional to the level of GSH in the vessels. **P<0.001 versus non-ischemic. (n = 5) (C) Ischemic tissues were probed with CMFDA for 30 min. Tissues were then homogenized and centrifuged at 6500 g for 5 min. The supernatants were then read at excitation/emission of 485/515 nm. Values obtained were normalized with the weight of the tissues. **P<0.001 versus non-ischemic. (n = 5).

Different ROS parameters like superoxide, hydrogen peroxide, peroxynitrite and lipid peroxidation. were evaluated in the ischemic vessels of chick embryo (Fig. 1) using different redox probes. Mapping of lipid peroxidation was carried out using the thiobarbituric acid (TBA) protocol. A higher level of lipid peroxidation was observed in the proximal areas in relation to the ischemic zone while lipid peroxidation level reduced in distal areas (Fig. 2A,2B). Image analysis and colorimetric measurement of superoxide in ischemic tissues demonstrated 40% and 42% increase in superoxide level respectively (Fig. 3). Measurement of hydrogen peroxide using amplex red depicted that ischemia attenuated hydrogen peroxide production by 25% (image analysis) and 27% (fluorimetric measurement) respectively (Fig. 4). Nitric oxide (NO) level in the ischemic vessels was measured using diamino fluorescein diacetate (DAF-2DA) fluorescence probe. There was no significant difference in NO production between ischemic and non-ischemic vessels was observed (Fig. 5A,5B). Peroxynitrite generation in the ischemic vessels was measured using dihydrorhodamine (DHR123) fluorescence probe. Partial ischemia elevated the peroxynitrite level in tissue by 33% compared to non-ischemic controls (Fig. 5C). Image analysis and fluorimetric measurement of free thiol in ischemic tissues using 5-chloromethylfluorescein diacetate (CMFDA) elaborated 48% and 42% reductions respectively in free thiol level under partial ischemia treatments (Fig. 6).

### Partial ischemia dependent higher HIF-1α release induced the neo-vascularization in the adjacent vessels

**Figure 7:**
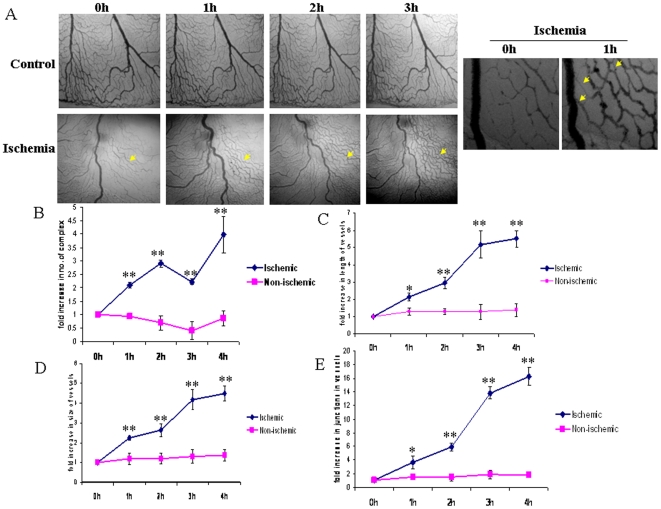
Studying the angiogenesis pattern in vessels adjacent to ischemic area. (A) Angiogenesis pattern of the vessels adjacent to the ischemic area were followed for 3 h. The images of the vessels adjacent to the ischemic area were taken with Nikon Cool Pix camera adapted to a stereo microscope. Images were converted to gray scale and presented. Inset showed the magnified images from ischemia group which revealed formation of new vessels in the area. Yellow arrows showed the formation of new vessels in the area. Images are the representative of 5 individual experiments. (B) Analysis of the images were carried out using Angioquant software. Number of separate vessel complexes was analyzed by the software for different time point. Data presented as fold increase with time. **P<0.001 versus respective time point under non-ischemia. (C) Similarly, the software calculated the length of the vessels in different time of incubation. Data presented as fold increase with time. *P<0.05 and **P<0.001 versus respective time point under non-ischemia. (D) The size of the vessels was calculated by the Angioquant software. Data presented as fold increase with time. **P<0.001 versus respective time point under non-ischemia. (E) The number of junctions in the vessels were calculated by the software and plotted. *P<0.05 and **P<0.001 versus respective time point under non-ischemia.

HIF-1α, a hypoxia driven factor, is a well known pro-angiogenic factor as reported earlier [5]. We evaluated the concept and studied the angiogenesis pattern of the vascular bed adjacent to the ischemic area. Analysis of the vascular bed images using Angioquant software revealed that ischemia elevated the neo-vascularization in chick embryo vascular bed in a time dependent manner. Analysis of number of vessel complexes, vessel length, vessel size and junctions revealed 4, 5.5, 4.5 and 11 fold increase respectively after the ischemia treatment (Fig. 7). Increase in vascularization can also be due to backfilling of initially occluded vessels. However, critical analysis of the images from ischemic vessels showed that the vessels were formed newly from the existing vessels and thus can be concluded that increase in the vessel density is due to the neo-angiogenesis in that area.

### Partial ischemia in chick embryo vessels elevated the expression level of HIF-1α in a time-dependent manner

**Figure 8:**
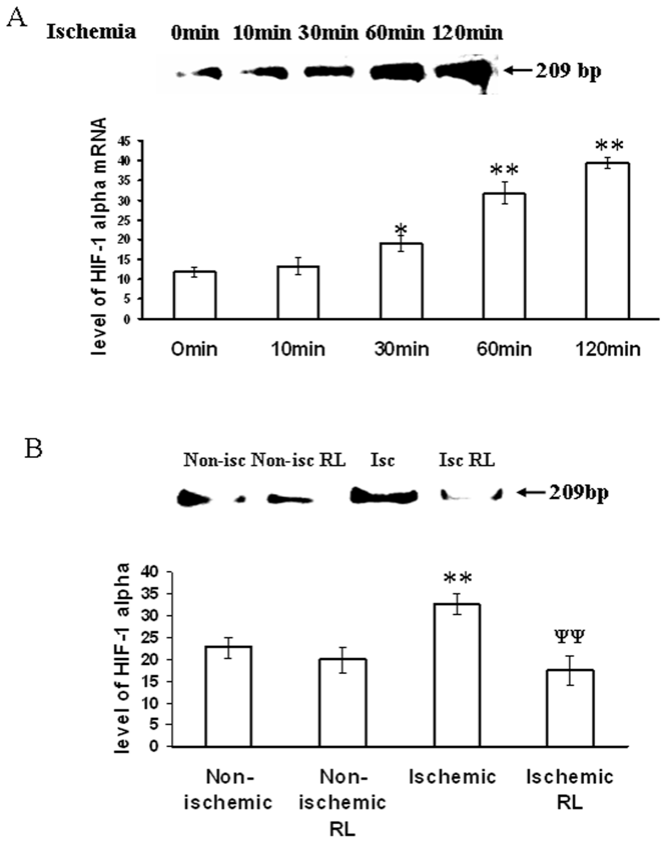
RL based recovery of GSH level in ischemic vitelline vascular tissue. (A) Level of free thiol in ischemic tissues treated with and with out RL were measured using CMFDA. Ischemic vessels were incubated with 100 µM of RL during the period of treatment. Next, the ischemic tissues were incubated with CMFDA for 30 min and imaged with DP71 CCD camera. Images are the representative of 5 individual experiments. (B) The fluorescent intensities of the images were calculated using Adobe Photoshop 7.0 and plotted. Fluorescent intensities were considered as directly proportional to the level of free thiol in tissues. **P<0.001 versus non-ischemic and ^ψψ^P<0.001 versus ischemia. (n = 5).

HIF-1α expression was used as a biochemical marker to quantify the level of ischemic stress in tissues. Relative expression of HIF-1α in ischemic vessels was measured using quantitative RT-PCR technique. The vessels were ligated and incubated for different durations ranging from 0 to 120 min. Quantitative analysis of the band intensities revealed a dose dependent increase in HIF-1α expression in the ischemic vessels. HIF-1α expression level was elevated by 1.5, 2.6 and 3.3 fold after 30, 120 and 240 min of ischemia treatments respectively. However, no significant increase in HIF-1α expression was observed in the ischemia-treated tissues ligated for 10 min (Fig. 8A). This data suggested that partial ischemia was successfully improvised in the chick embryo vascular bed using the strategy of right vitelline artery ligation model.

### Ranolazine (RL) treatment attenuated HIF-1α expression and protected the free glutathione (GSH) levels in ischemic vessels

**Figure 9:**
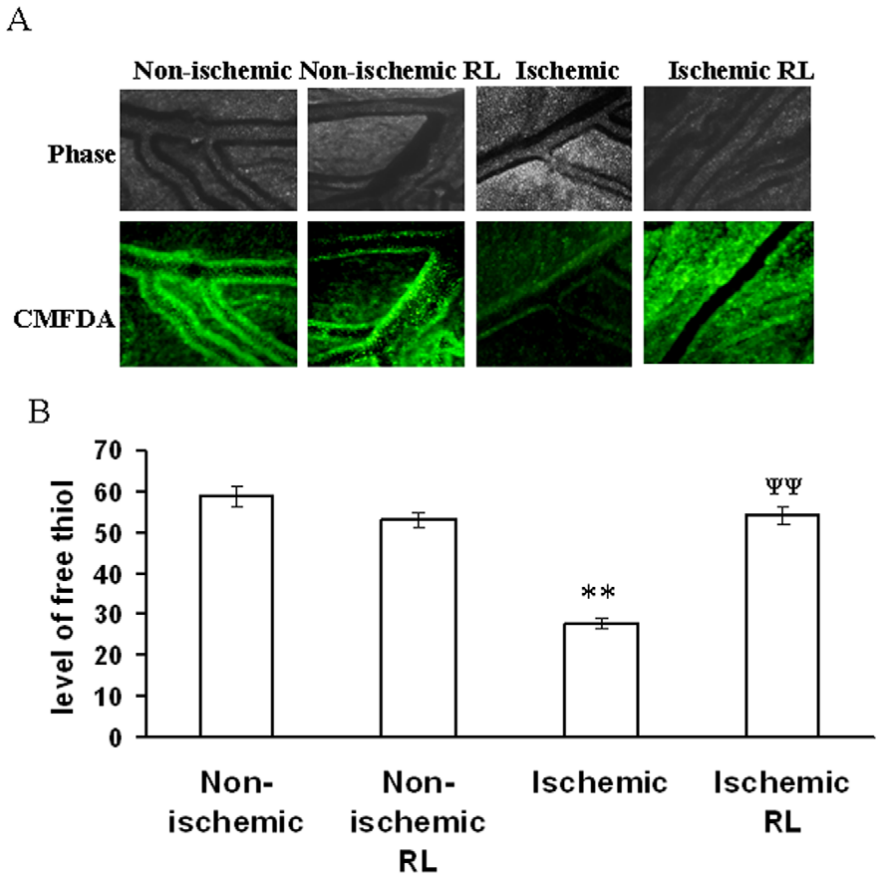
HIF-1α expression level in ischemic vitelline vascular tissue. (A) A time dependent HIF-1α expression in the ischemic tissues were quantified using quantitative RT-PCR technique. After treatment mRNA was isolated from the tissues using RNA extraction kit followed by quantitative RT-PCR. Total extracted RNA was quantified by running a 2%agarose gel containing ethidium bromide. Total RNA loading for cDNA conversion was then normalized from the band luminosity of total RNA from different samples. Amplified products using specific primers for HIF-1α was then run on 2% agarose gel and visualized. The band luminosity was then quantified and plotted. Band intensities were considered as directly proportional to the HIF-1α mRNA level in the tissues. Images are the representative of 3 individual experiments. *P = 0.012 and **P<0.001 versus 0 min time point. (n = 3) (B) HIF-1α expression level was measured in ischemic vascular bed with RL treatment. RL was treated during the period of ischemia. RL attenuated ischemia induced HIF-1α expression. Images are the representative of 3 individual experiments. **P<0.001 versus non-ischemia and ^ψψ^P<0.001 versus ischemia. (n = 3).

RL, a well known anti-ischemic drug was used in the present study to recover ischemia-dependent tissue injury. Free glutathione (GSH) being the quencher of cellular ROS protects the cells and tissue from ROS mediated damage. In the present study, we measured the level of GSH using CMFDA fluorescence probe. The result depicted that ischemia attenuated the cellular GSH level by 53% while RL administration recovered the GSH level by 52% in the ischemic vessels of chick embryo (Fig. 9A,9B). The expression level of HIF-1α was measured in the ischemic vessels under RL treatment using quantitative RT-PCR technique. Ischemia elevated the level of HIF-1α expression by 38% while RL administration attenuated HIF-1α expression in ischemic vessels by 48% (Fig. 8B). However, RL did not affect the expression level of HIF-1α and level of GSH under non-ischemic condition (Fig. 9A,9B). This data elaborated that RL administration protected chick embryo vessels from ischemia induced damage.

### Partial ischemia interferes with cellular morphology of EC grown on the ischemic bed

**Figure 10:**
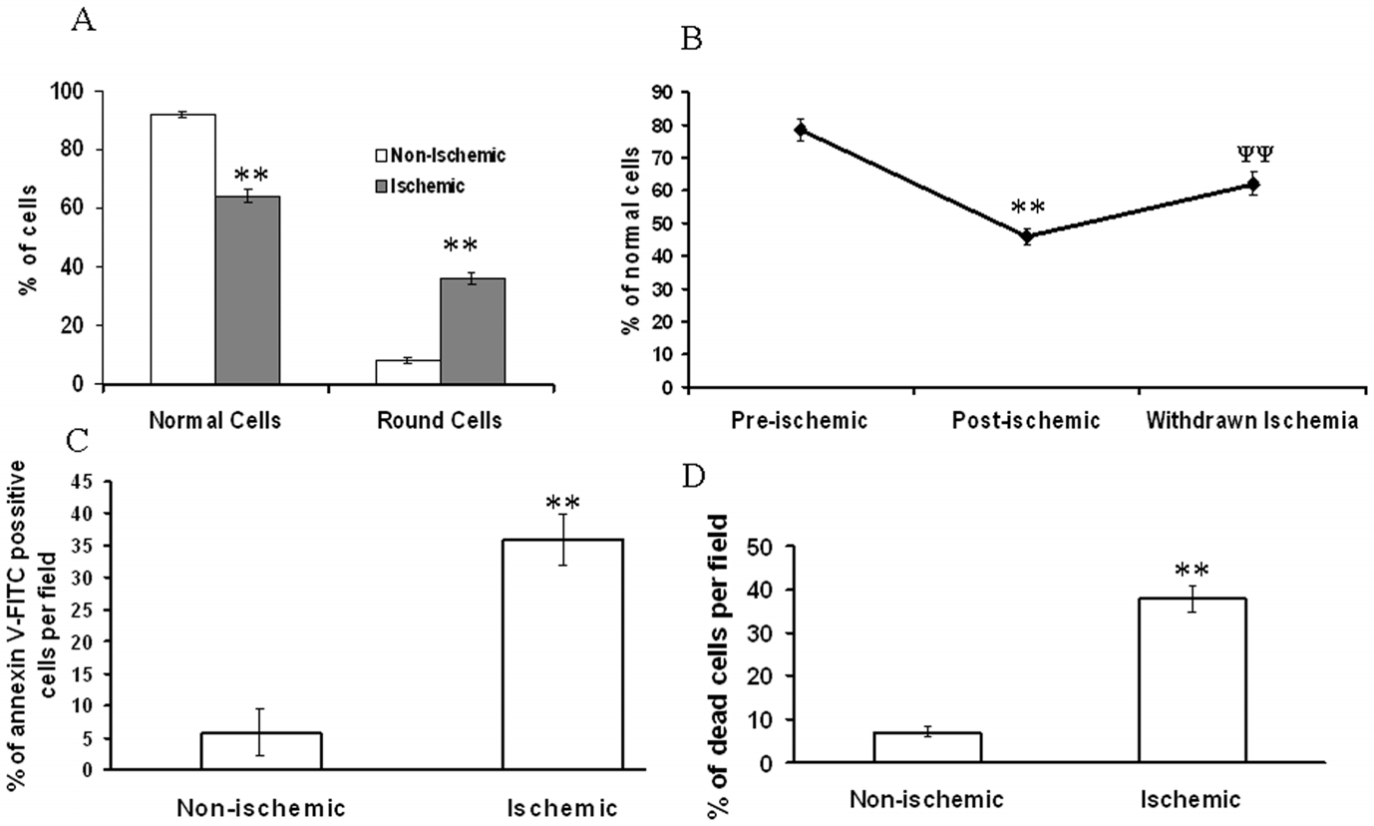
Effect of ischemic vascular bed driven secondary ischemia on EC. (A) The number of round and normal cells were counted from the bright field images. Data were normalized by counting the total number of cells per field and presenting it as percentage of cell. **P<0.001 versus non-ischemic. (n = 7) (B) Number of normal cells were counted from each field and plotted as percentage of normal cells. Ischemia induced reduction in normal cell number was calculated while reversibility was observed when the cells kept back on normal vessels. **P<0.001 versus preischemic and ^ψψ^P<0.001 versus postischemic. (n = 7). (C) Apoptosis was measured in ischemia treated EC using annexin-V FITC detection kit. The total number of cells and the number green positive cells per field were counted and plotted. Images are the representative of 3 individual experiments. **P<0.001 versus non-ischemic. (n = 3) (D) Cellular viability under ischemia treatment was measured using trypan blue dye. Trypan blue stained cells were taken as dead cells. The total number of cells and the number green positive cells per field were counted and plotted. Images are the representative of 7 individual experiments. **P<0.001 versus non-ischemic. (n = 7).

Morphological analysis of EC grown on the ischemic bed was carried out to evaluate the effect of ischemia on cellular health. EC grown on collagen-coated cover slips were treated under partial ischemia by incubating it on the top of ischemic vascular bed. Images showed a higher number of round cells under ischemic condition. This data demonstrated that ischemic vascular bed driven secondary ischemia in EC interfered with cellular morphology (Fig. 10A).

To verify the effect of withdrawing ischemia on cellular morphology, we studied the morphology of the cells by placing the ischemia treated cells on non-ischemic vascular bed. Ischemia reduced the number of normal cells by 32% while placing back the EC on a non-ischemic bed reversed back the number of round cells by 19% (Fig. 10B). This data suggested that ischemia dependent cellular damage can be partially reversed by withdrawing ischemic stress.

### Ischemic vascular bed driven secondary ischemia compromised cellular viability and induced cellular apoptosis

Analysis of apoptosis in EAhy926 cells under partial ischemia treatment was carried out using annexin V-FITC apoptosis detection kit. Result depicted that partial ischemia treatment is apoptotic to cells thus facilitating the binding of annexin V-FITC to the outer part of the plasma membrane (Fig. 10C). Partial ischemia induced endothelial apoptosis by 8 fold compared to non-ischemic control (Fig. 10C). Cellular viability of EC under partial ischemia treatment was assayed using trypan blue dye. Images of the cells revealed that partial ischemia increased the number of trypan blue stained cells by 8 fold (Fig. 10D).

### Ischemic vascular bed elevated ROS generation in EC

**Figure 11:**
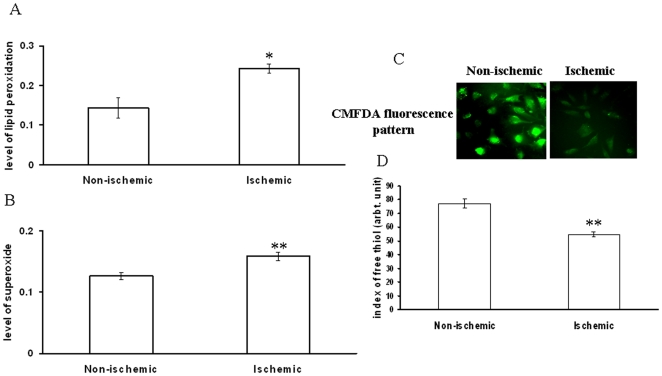
Level of different reactive oxygen species and GSH in ischemic EC. (A) Level of lipid peroxidation in ischemic vascular bed treated EC was quantified using TBA protocol. EC were treated under ischemia for 2 h followed by processing as mentioned in the materials and methodssection. Data were normalized by counting the number of cells using hemocytometer. Elevated lipid peroxidation was noted under ischemia treatment. *P = 0.012 versus non-ischemic. (n = 5) (B) Superoxide production by ischemia treated EC was measured using NBT. Cells were treated under ischemia for 2 h followed by incubation with NBT for another 2 h. The formed formazen dye was extracted from the cells as mentioned in methodology. Data were normalized by counting the number of cells using hemocytometer. **P<0.001 versus non-ischemic. (n = 5) (C) Ischemia treated EC were probed with CMFDA to measure the level of GSH in the cells. Cells were treated with ischemic vascular bed driven secondary ischemia for 2 h followed by probing with CMFDA for 30 min. Images were acquired by DP71 camera adapted to an Olympus IX71 microscope. Images are the representative of 5 individual experiments. (D) The green luminosity from the CMFDA probed fluorescent images were measured using Adobe Photoshop version 7.0. Green luminosity of 40 different cells from 5 different sets of experiment were measured and plotted. Ischemia reduced the level of free thiol in EC. **P<0.001 versus non-ischemic. (n = 5).

Two different ROS parameters were studied in EC under partial ischemia treatment. Superoxide and lipid peroxidation level in cells were measured after incubating EC on ischemic vascular bed using nitro blue tetrazolium (NBT) and TBA protocols respectively. Partial ischemia promoted superoxide generation in EC by 27% while the level of lipid peroxidation under partial ischemia treatment was elevated by 40% compared to non-ischemic control (Fig. 11A,11B). This data suggested that ischemic vascular bed improvised secondary ischemia in EC that in turn elevated ROS level in EC.

### Partial ischemia reduced the GSH concentration in EC

The level of free thiol in EC was measured using CMFDA fluorescent probe. Partial ischemia reduced the level of GSH in EC by 32% (Fig. 11C, 11D).

### Anti-ischemic drug treatments improved the cellular health condition and preserved the GSH level under partial ischemia

**Figure 12:**
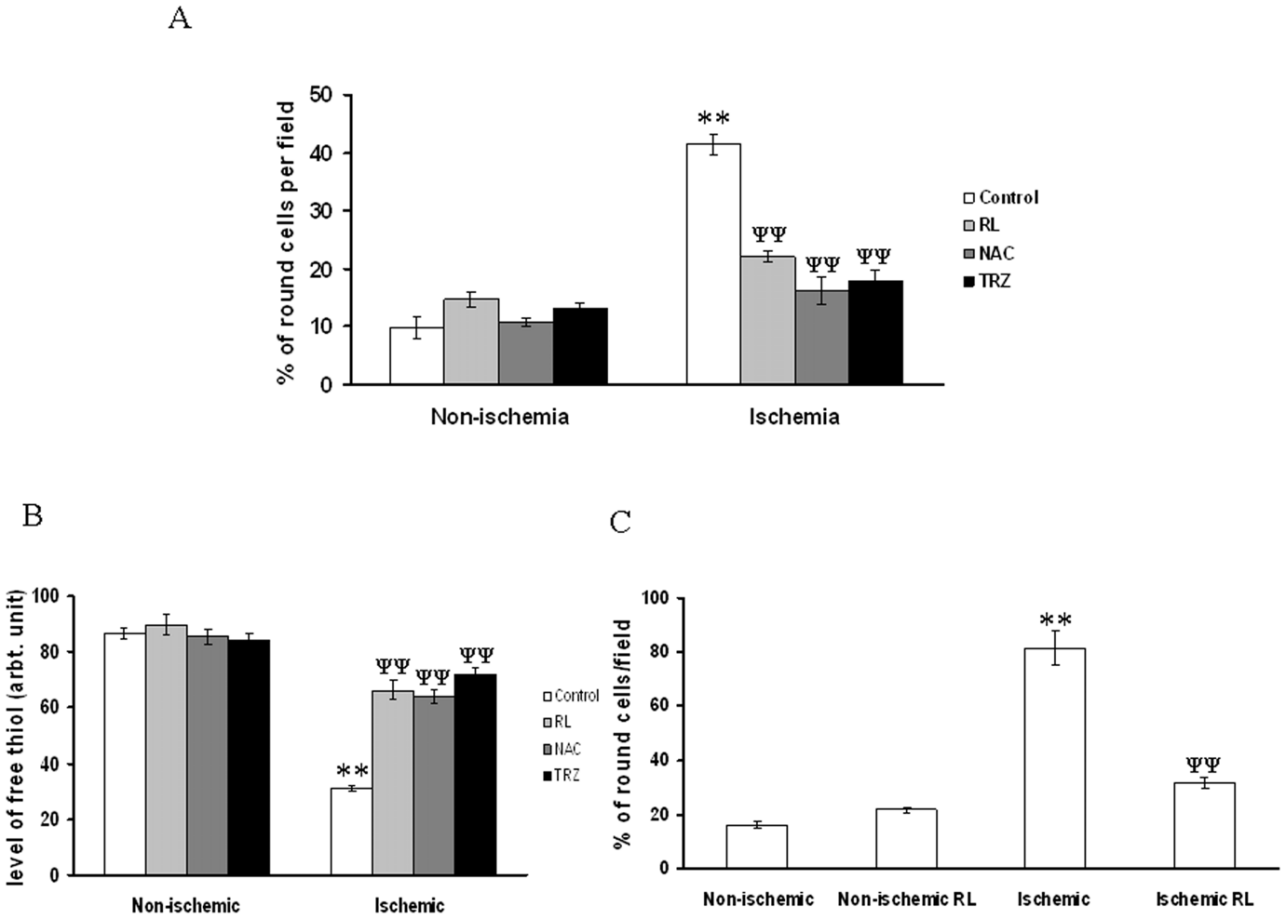
RL driven recovery of ischemia treated EC activity. (A) Number of round cells per field were counted from the bright field images. Data were normalized by counting the total number of cells and presenting as percentage of round cells per filed. RL, NAC and TRZ attenuated ischemia driven rounding up of the cells. No effect of RL, NAC and TRZ were observed under non-ischemic condition. **P<0.001 versus non-ischemic and ^ψψ^P<0.001 versus ischemia. (n = 7) (B) Green luminosity from the images was measured using Adobe Photoshop version 7.0. Fluorescence intensities of 40 cells from 5 different sets of experiments were calculated and plotted. **P<0.001 versus non-ischemic and ^ψψ^P<0.001 versus ischemia. (n = 5). (C) Number of round cells was counted per field from the bright field images. Data were normalized by counting the total number of cells in each field and plotting as percentage of round cells per field. ^**^P<0.001 versus non-ischemic and ^ψψ^P<0.001 versus ischemic. (n = 7).

To evaluate the protecting role of antioxidant and anti-ischemic drug, we performed morphology counting and GSH measurement experiments in ischemia treated EC in the presence and absence of anti-ischemic drugs. Results depicted that ischemia induced rounding up of EC while RL, N-acetyl cysteine (NAC) and trimetazidine (TRZ) treatments protected cells from ischemia induced stress. However, non-ischemic cells were insensitive to RL, NAC and TRZ treatment (Fig. 12A). GSH measurement in ischemia treated EC using CMFDA elaborated that ischemia reduced the level of GSH while RL, NAC and TRZ potentiate the cells to maintain normal GSH concentration under ischemia (Fig. 12B). However, RL did not show any effect on the level of GSH in non-ischemia treated EC. Morphology of H9C2 cells under ischemia was studied with and without RL administrations. Ischemia promoted the rounding up of H9C2 cells while administration of RL protected the cells from ischemia induced rounding up (Fig. 12C). However, non-ischemic cells were insensitive to RL administration.

### Partial ischemia induced HIF-1α expression in EC while RL attenuated ischemia induced over expression of HIF-1α

**Figure 13:**
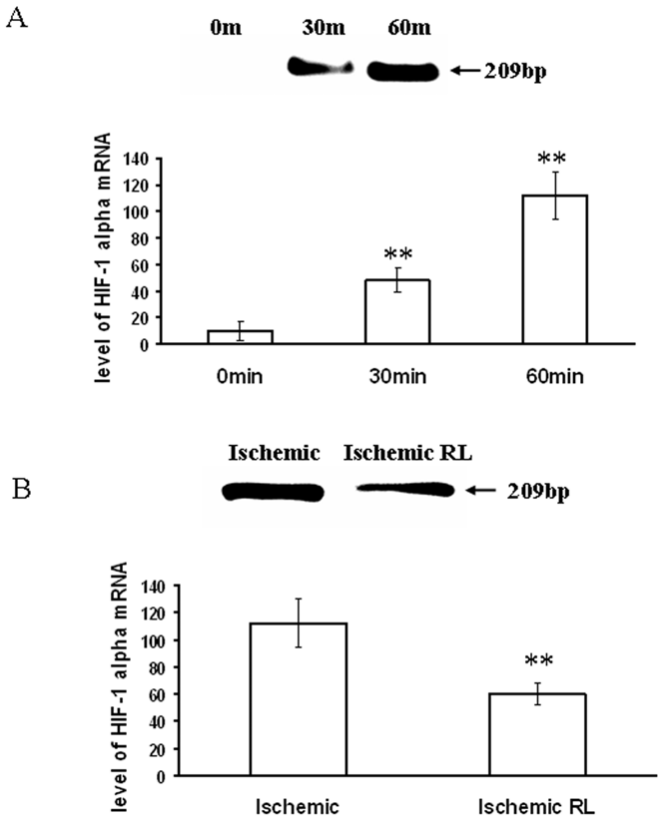
HIF-1α level in ischemic EC. (A) HIF-1α production was measured in EC treated for different time under ischemia. Cells were grown on collagen coated cover glasses and placed on the top of the ischemic vascular bed of chick embryo. RNA was isolated from the ischemia treated EC using RNA extraction kit. Loading of total RNA for cDNA conversion was normalized by running a gel for total RNA and calculating the band intensities for each set. After amplification, the band intensities from the representative images were calculated and plotted as a graph. Ischemia induced HIF-1α expression in EC in a time dependent manner. **P<0.001 significantly different than 0 min. (n = 5) (B) RL was administered to evaluate its effect on ischemia induced HIF-1α expression by EC. EC grown on collagen coated cover glasses were placed on ischemic chick embryo vascular bed followed by RNA extraction from the EC using RNA extraction kit. Loading of total RNA for cDNA conversion was normalized by running a gel for total RNA and calculating the band intensities for each set. RL attenuated ischemia induced HIF-1α expression by EC. **P<0.001 versus ischemic. (n = 5).

To evaluate the protective effect of RL, HIF-1α mRNA expression was measured in RL treated EC under partial ischemia treatments. Quantitative RT-PCR data elaborated that ischemia induced HIF-1α expression in EC by 6 and 11 fold in 30 min and 60 min ischemia treated cells respectively (Fig. 13A). Administration of RL blocked ischemia induced HIF-1α expression by 1.8 fold (Fig. 13B).

### Ischemic vascular bed mediated partial ischemia in caprine heart elevated the level of creatine phospho kinase-MB (CK-MB) activity in the heart tissues

**Figure 14:**
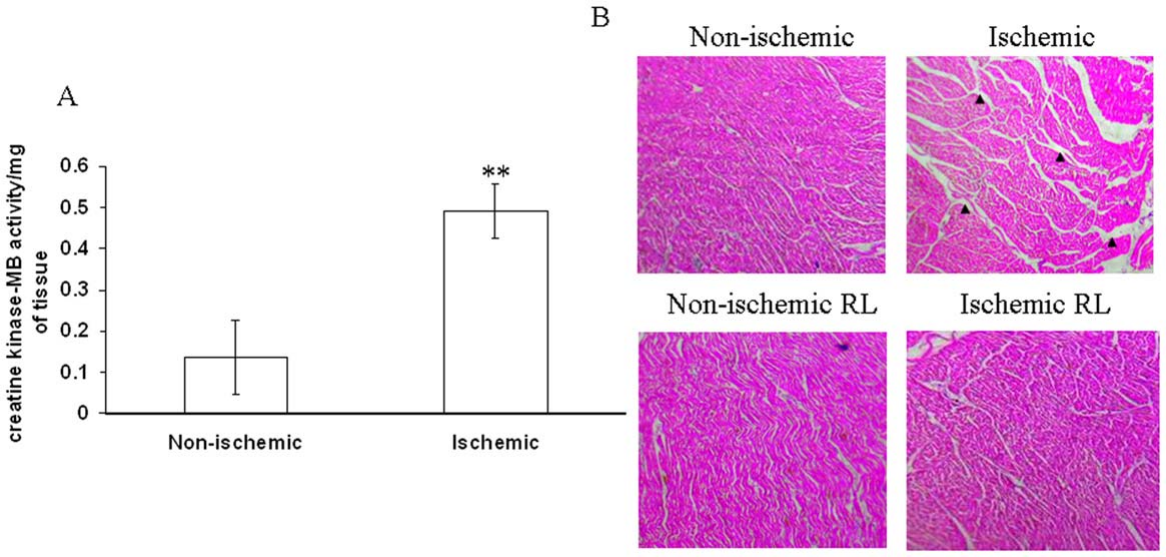
Effect of ischemic vascular driven secondary ischemia on caprine thoracic goat heart strip. (A) Caprine thoracic goat hearts were obtained from government authorized slaughter houses. Thin section of the hearts were made using surgical blade and placed on the top of ischemic vascular bed of chick embryo and incubated for 2 h. Next, the heart strips were homogenized in HBS containing protease inhibitor and centrifuged at 10,000 g. The CK-MB activity was then measured in the supernatant and the values obtained were plotted after normalizing with the weight of the tissues. Ischemia induced the CK-MB activity of the heart muscle. **P<0.001 versus non-ischemic. (n = 3) (B) Heart strips were treated under ischemia and fixed with 10% formalin. Next, the strips were embedded in paraffin wax and sectioned. The thin sections were stained with hematoxin-eosin stain and imaged with Nikon Cool Pix camera adapted to a stereo microscope. Ischemia induced edema and an elevated intra-mascular gaps as showed by the black arrows. Images are the representative of 5 individual experiments.

To evaluate the efficacy of the present model on creating secondary ischemia in heart tissues, we incubated the caprine heart tissue on the ischemic vascular bed. To confirm ischemia in the heart tissues, we measured the activity of CK-MB, a known marker of myocardial ischemia. Biochemical analysis of the heart tissue sample depicted that ischemic vascular bed mediated ischemia in caprine heart tissues elevated the enzyme activity of CK-MB by 72% (Fig. 14A). Elevation of CK-MB activity in caprine heart elucidated that ischemic vascular bed of chick embryo successfully induced secondary ischemia in caprine heart tissues.

### Partial ischemia caused edema and higher intra-muscular gap in caprine heart

Caprine heart tissues were incubated on the ischemic vascular bed and fixed with 10% formalin. Parafilm wax embedded tissue sections were stained using hematoxylin and eosin stains. Images of the sections depicted that ischemic vascular bed promoted edema and an elevated intra-mascular gaps in caprine heart tissue (Fig. 14B). This data suggested that chick embryo ischemic vascular bed induced secondary ischemia in caprine heart tissue.

## Discussion

Different animal models are available for tissue specific ischemia such as embolic middle cerebral artery occlusion and endovascular filament middle cerebral artery occlusion. Middle cerebral artery occlusion is achieved by injecting particles like blood clots or artificial spheres into the carotid artery of animals as an animal model of ischemic stroke. Thromboembolic MCAo is achieved either by injecting clots that were formed *in vitro* [6] or by endovascular instillation of thrombin [7]. The thromboembolic model is closest to the pathophysiology of human cardioembolic stroke. But, the fundamental problem in ischemia research is the lack of easy to develop routine research models, which should offer a regular testing platform for routine research and drug testing for ischemia. The present study focused to develop a partial ischemia model by using vascular bed of 96 h grown chick embryo. The present work aimed to develop a basic ischemia model instead tissue or organ specific one, which would cater the scientists in academic and industry to understand the fundamental aspects of ischemia implications and will also help in faster screening of anti-ischemic drugs. Although chick embryo never been used before for creating ischemia, efforts were made to develop chick embryo based flow-manipulation model for shear stress associated research [8]–[10]. Hogers *et al*., 1999 have taken two approaches to manipulate the blood flow in chick embryos [8]. First, one of the three major vitelline veins was ligated and secondly these vitelline veins were ligated permanently with a microclip until cardiac septation was completed. This venous clip model is one of the major approaches to control the blood flow in the vascular bed of chick embryo [8], [9]. However, these studies are directed towards evaluating the consequences of low blood flow on embryo development and cardiovascular malformations while the present study aims to develop an *ex vivo* partial ischemia model by using a similar approach to control the blood flow in chick embryo vascular bed. We ligated the right vitelline artery of chick embryo that blocks the blood-flow of corresponding areas of vascular bed in the chick embryo.

Work of Lee *et al*., 2000 and different other groups suggested that an early stage elevation of HIF-1α expression under myocardial ischemia is a protective measure by the cell to protect itself from the ischemia induced damage [11]–[16]. We measured the level of HIF-1α expression in the ligated chick embryo model by considering it as one of the early markers of ischemic tissue damage, and we also found that HIF-1α expression level elevated in affected vascular bed and also in cells cultured on the ischemic vascular bed. The work of Yamamoto *et al* (1983) demonstrated that lipid peroxidation plays a major role in ischemia driven cellular damages [17]. Works of different groups have also reported that a higher level of lipid peroxidation during ischemia and ischemic reperfusion causes cellular damage and cell death [17]–[19]. Yamamoto *et al*., 1983 have shown that administration of α-tocopherol, a ROS scavenger and a blocker of lipid peroxidation improves the cellular health under ischemia [17]. Similarly EC incubated on the ischemic vascular bed of chick embryo evoked a higher level of lipid peroxidation indicating that primary ischemia in the vascular bed spreads secondary ischemia in the cells and tissues grown on the affected vascular bed.

ROS generation during ischemia is a consequence of ischemia mediated tissue damage [20]–[22]. The burst of ROS during ischemia reperfusion is considered as one of the key factors in the development of myocardial stunning after regional ischemia [23], [24]. Generation of ROS during ischemia [25]–[27] are considered as an important factor in the development of cardiac preconditioning [28], [29]. Work of Becker *et al*., 1999 concluded that significant superoxide generation occurs during ischemia before reperfusion from the ubisemiquinone site of the mitochondrial electron transport chain [25]. All together these studies signify that ischemia induced ROS generation by cellular systems plays a major role in ischemia mediated tissue damage. Similarly, in the present study, we observed a higher level of superoxide generation in both ischemic tissues and in cells treated with the ischemic vascular bed. However, we observed that blocking of right vitelline artery suppressed hydrogen peroxide production. Hydrogen peroxide is known to confer protection to ischemic tissues. The work of Yada *et al*. (2006) showed that endogenous hydrogen peroxide, in cooperation with NO, plays an important cardioprotective role in coronary ischemia reperfusion injury *in vivo* [30]. It has been reported that hypoxia activates catalase pool in the cells. A classic work by Oshino *et al*. (1975) demonstrated that the rate of hydrogen peroxide production in the “haemoglobin-free”, “non-circulatory” perfused liver of rats decreased to almost half in the livers of starved and phenobarbital-pretreated rats [31]. Enhanced superoxide generation and simultaneous fall in hydrogen peroxide in the present partial ischemia model indicates a reduced dismutation of superoxide by its classical SOD pathway, which is the prime source of hydrogen peroxide. In stead superoxide possibly interacts with NO to form peroxynitrite that is evident from the results of the present study. Work of Scaduto *et al*., 1988 depicted that the content of GSH in the rat declined to 40% of control values during 35 min of renal artery occlusion [32]. In the present partial ischemia model, the level of GSH, a potent quencher of ROS was suppressed. We deem that low GSH level under partial ischemia leads to higher ROS generation that caused damage to EC and cardiomyofibroblast cells as observed in the present study. The result of present work further ascertains that deteriorating functional parameters of the EC is the combinational effect of ischemia and associated ROS generation from the ischemic vascular bed.

RL, a specific inhibitor of late sodium current reduces sodium overload and hence ameliorates disturbed ion homeostasis in cardiac cells [33]–[39]. In the present study we administered RL as an anti-ischemic drug and evaluated its activity on different functional and biochemical parameters of the cells and tissues under ischemia. As observed by different groups, present study also suggests that RL can protect the cells and tissues from secondary ischemia developed by incubating the cells on ischemic vascular bed of chick embryo. The results offered another line of support that the present model is capable of inducing ischemia in cells and tissues. Reports from earlier findings described the protective role of NAC after ischemia or ischemic reperfusion injury [40], [41]. In addition to RL, we used these two well known anti-ischemic drugs NAC and TRZ [42], [43] to elucidate the efficacy of these drugs in recovering ischemia based cellular injury in the present model. NAC and TRZ both protected EC from ischemic vascular bed driven secondary ischemia.

CK-MB activity has been shown to increase in certain patients with ischemic stroke, sub-arachnoid hemorrhage and head trauma in the absence of any clinically evident acute coronary syndrome [31], [44]–[47]. The temporal pattern of elevation was typically gradual and sustained for several days, unlike myocardial infarction, in which CK-MB peaks and falls within the first 24 h of coronary artery occlusion [48]. Similarly, our study depicted that CK-MB activity is high when the caprine heart tissue sample was incubated on the ischemic vascular bed of chick embryo. As reported earlier by Zhai *et al*., 2000, we also observed a similar histology pattern of the ischemic heart tissue sample where marked interstitial edema and evidence of intramascular gaps were observed when incubated under ischemic vascular bed driven ischemia [49]. These observations demonstrate that ligation mediated partial ischemia in chick vascular bed could create secondary ischemia in the caprine heart tissues as well.

The ligation of right vitelline artery in chick embryo causes partial ischemia in the surrounding vascular bed, which can further be used to create secondary ischemia in cells and other tissues under *in vitro* condition. The present model offers some unique features for routine ischemia related drug testing such as 1) less ethical issues 2) easy to develop model 3) real time observation of physiological parameters such as blood flow and angiogenesis and 4) culturing of cells and tissues of one's choice on the ischemic bed makes the model very plastic. However, the present model also has limitations in its present form. The limitations are 1) First of all, the model cannot be tissue or organ specific as it is possible in animal models. 2) Secondly, the model cannot produce intermittent ischemia or ischemic reperfusion injury, which is prevalent in human. 3) Unlike adult tissues, the avian embryo during early stages of development obtains oxygen through direct diffusion into the tissues (until day 5–6). In subsequent stages the oxygen diffuses into blood circulating through the extra-embryonic chorioallantoic membrane. However, due to blockade in artery, blood flow to that specific area is blocked which will partially hinder with the diffusion properties of free oxygen to the vessels. Therefore, a partial blockade in both oxygen and nutrient diffusion was obtained in the present model and thus termed as partial ischemia model.

Particularly, during dynamic exercise, normal coronary arteries dilate, whereas stenotic arteries constrict. This exercise-induced vasoconstriction has been associated with the occurrence of myocardial ischemia and has been believed to be the result of endothelial dysfunction, with a reduced release or production of NO, increased sympathetic stimulation, enhanced platelet aggregation with release of thromboxane A2 and serotonin or a passive collapse of the disease-free wall segment within the stenosis (the Bernoulli effect) or a combination of any of these [50]. It is not possible to exert exercise like effects on the present model of ischemia. However, work is in progress in our laboratory to modulate the blood flow in a specified rhythm specifically in a defined vessel in the vascular bed by using macro-manipulator to achieve the intermittent and modulated ischemia along with reperfusion effects.

The results of the present work also suggested that anti-ischemic efficacy of any drug can be evaluated in the present model before studying the drug in any other animal models. Therefore, the present model can be used as an additional model along with other *in vivo* ischemia models to reduce animal experimentations.

## Materials and Methods

### Materials

Dulbecco's modified Eagle's medium (DMEM) was purchased from PAN-Biotech GmbH, Am Gewerbepark, Aidenbach. Fetal bovine serum was from Invitrogen Life technologies. Diamino fluorescein diacetate, 5-chloromethylfluorescein diacetate, amplex red, dihydrorhodamine 123 (DHR) was purchased from Moleculer Probe, Oregon, USA. RNA isolation spin prep kit was from Medox Inc., India. cDNA Mulv reverse transcriptase and taq polymerase was purchased from New-England Biolabs, MA and Finnzymes, Espoo, Finland respectively. RL [N-(2,6-dimethylphenyl)-2-[4-[2-hydroxy-3-(2-methoxyphenoxy) propyl]piperazin-1-yl]acetamide] and TRZ was purchased from Sigma Chemicals, St. Louis, MO, USA. Nitro blue tetrazolium and NAC was purchased from Hi-Media, Bangalore, India. All other chemicals were of reagent grade and were obtained commercially.

### Cell culture

Immortalized endothelial hybrid cell lines, EAhy926 were gifted from Dr C.J.S. Edgell. H9C2 myofibroblast cells were purchased from NCCS, Pune, India. Both the cells were maintained and cultured in DMEM supplemented with 10% FBS (v/v) and 1% penicillin (w/v) and streptomycin (w/v).

### Animals

Fertilized white Leghorn chick (*Gallus domesticus* L.) eggs were obtained from Poultry Research Station, Nandanam, Chennai, TN, India and incubated at 37–38°C, with the blunt end up and at a relative humidity of 70–80%. Caprine heart samples were collected from the government authorized slaughter houses.

### Creating partial ischemia on chick embryo vascular bed using artery ligation model

In the early stages of chick embryo development, two distinct circulatory systems are established in an embryonic system, one for the embryo itself maintaining the blood circulation inside the embryo body and another vitelline system extending into the egg sac, which helps the embryo to get nutrients from the yolk and diffused oxygen from the air. Blocking the circulation of any of the vitelline artery can interfere with nutrient and oxygen diffusion to the blood thus can create a partial ischemia. In the present study, ischemia was created in the vascular bed of chick embryo by using a modified protocol as mentioned by Vos *et al*., 2003 where they studied the hemodynamics due to blockade in right vitelline vein [51]. 96 h grown chick embryos were broke open in a sterile glass bowls (Fig. 1A). Only embryos that showed no bleeding or deformities were selected. The material was subdivided into artery ligated embryos and control embryos. In the ligation group, a small incision in the yolk sac membrane was made. Subsequently, the right vitelline artery was blocked using a surgical suture (Fig. 1B). Cessation of blood flow proximal to the ligation was confirmed under stereo microscopic surveillance. The sutures were made up of 0.1 mm thick bio-friendly and biodegradable surgical thread. All experiments were performed *ex vivo*. During the experiments, all eggs were placed on a thermo element of 37°C/humidity and covered with a sterile glass lead.

### Preparation of the tissue samples for measuring HIF-1α and different ROS parameters

After partial ischemia, vascular bed of the chick embryo was surgically incised out using a sterile surgical scissor. For mapping lipid peroxidation, the whole vascular bed along with the embryo was taken off from the yolk sac and placed in Petri plate containing PBS buffer. Next, the vascular bed was dissected out from the embryo into six equal sections. At the end of the dissection, the embryo left untouched while the whole vascular bed were sectioned to six equal portions. The left non-ligated portion was taken as our control tissue. Tissues were then washed 3–4 times with HBS in order to wash out all the yolk material and the blood from the vessels. Care was taken to avoid any damage to the vascular bed of chick embryo. Next, respective tissue samples were processed according to the experimental need.

### Treatment of different cells under ischemia using the ischemic vascular bed of chick embryo

Work of different groups showed that mammalian cells can be grown easily using the hen's yolk as growth media [52], [53]. EAhy926 cells and H9C2 cells were grown in collagen coated cover glasses for overnight at 37°C/5%CO_2_. Equal numbers of cells were seeded on cover slips according to the experimental need. Next, the cover glasses containing the cells were placed on top of the ischemic vessels thus allowing the cells to be in direct contact with the ischemic area.

### Measurement of free glutathione using CMFDA

Measurement of GSH was performed using the CMFDA fluorescence probe as mentioned else where [54]. Ischemia was created in the blood vessels by following the protocol as mentioned earlier followed by incubating the cells on the top of the ischemic vessels. For RL based recovery experiment, vessels were treated with RL during the period of ischemia while cells were pretreated with RL for 15 min followed by incubation on RL treated ischemic vessels. Both tissues and cells were washed with PBS and incubated with CMFDA for 30 min. Next, the unbound CMFDA was washed of by washing with PBS for 2 times. Fluorescence images of the CMFDA bound tissues and cells were taken using Olympus IX71 microscope adapted with a DP71 camera. Next, fluorescence intensities of the images were calculated using Adobe Photoshop version 7.0.

### Measurement of superoxide using NBT

Superoxide in ischemic vessels and cells were measured using NBT. Briefly, partial ischemia was created in the vessels by blocking the blood flow while the ischemic vascular bed was used to treat the cells under ischemia. Next, vessels and cells were washed with PBS and incubated with NBT for 2 h. For imaging, the vessels were washed with PBS and the images of the NBT stained vessels were taken with Nikon Cool Pix camera adapted to a stereo microscope. For colorimetric analysis of the sample, tissues and cells were homogenized and centrifuged at 6500 g to remove the debris. Absorption of the supernatant was taken in BioRad Elisa reader at 560 nm.

### Measurement of peroxynitrite using dihydrorhodamine

DHR123 was used to quantify the level of peroxynitrite in ischemic vessels and cells. After ligation of the vessels, the chick embryos were kept for 2 h and the vascular bed was incised out using a sterile scissor. Tissues were washed with PBS and incubated with the DHR123 probe for 1 h. After 1 h incubation, the tissues and cells were washed with PBS and homogenized with a glass homogenizer. Homogenized samples were then centrifuged at 6500 g for 5 min and the supernatant was read at excitation/emission of 500/536 nm.

### Hydrogen peroxide measurement using amplex red

H_2_O_2_ level in ischemic vessels of chick embryo was measured using H_2_O_2_ specific fluorescence probe, amplex red. Ischemic vessels were incised out and washed with PBS for 3–4 times. Next, the tissues were incubated with amplex red for 30 min. For imaging, tissues were washed with PBS for 2–3 times and fixed on glass cover slips and the images were taken under 4X objective of Olympus IX71 microscope. For fluorimetric analysis, tissues were homogenized and centrifuged. The reading of the supernatant was taken at excitation/emission of 570/585 nm.

### Nitric oxide measurement using DAF-2DA fluorescence probe

NO levels in the ischemic chick embryo vessels were measured using the DAF-2DA fluorescence probe. Ischemic vessels were excised out and washed with PBS. Next, the vessels were incubated with DAF-2DA and 1 mM L-arginine mixture for 20 min. After washing, the DAF-2DA probed vessels were fixed on the glass cover slide and imaged under 4X objective of Olympus IX71 microscope. The fluorescence intensity from the vessels was measured using the Adobe Photoshop version 7.0.

### Lipid peroxidation using TBA protocol

We evaluated lipid peroxides by measuring TBARS using a technique modified from Bromont *et al*., 1989 [18]. Ischemic vascular bed and cells were homogenized in 5vol ice-cold 0.05 M phosphate buffer (pH 7). 1 ml buffer solution and 1.5 ml trichloro-acetic (TCA)-thiobarbituric acid (TBA)-HCl reagent prepared in 0.85N HCL with 13.5% wt/vol TCA and 0.33% wt/vol TBA and deoxygenated by bubbling with nitrogen were added to 0.2 ml homogenate. Samples were then placed in sealed tubes containing nitrogen and were heated for 15 minutes in a boiling water bath. After cooling, 1 ml TCA 70% wt/vol was added, and the precipitate was removed by centrifugation at 2,500 g for 10 minutes. The fluorescence of the supernatant was measured on Varian Carry Eclipse Fluorescence Spectrophotometer at 553 nm with an excitation wavelength of 515 nm. The level of fluorescence was taken as an index of lipid peroxidation both in tissue and cellular systems.

### Cell viability assay using trypan blue

Viability experiment was performed following the protocol as mentioned earlier [55]. In brief, EAhy926 cells were grown to about 60% confluence in collagen coated cover slips. The cells were then subjected to ischemia treatment. Media containing trypan blue (0.4 mg/ml) was added to the cells and incubated for another 15 min. After incubation, the media was removed and PBS was added to all the wells. Random images were acquired using Nikon Cool Pix camera adapted to a Motic inverted microscope and the numbers of cells having a blue colored nucleus were counted from the images.

### Apoptosis measurement using annexin V-FITC apoptosis detection kit

EAhy926 cells were grown overnight on collagen coated cover slips and treated under partial ischemia using the ischemic vascular bed of chick embryo. Next, treated cells were processed using the protocol as supplied by the manufacturer (Merck, Calbiochem, EMD Chemicals Inc., Darmstadt, Germany). Fluorescence images of the cells were taken and the number of annexin V-FITC positive cells were counted per field.

### Angiogenesis pattern of the vascular bed of chick embryo adjacent to the ischemic area

HIF-1α is a known inducer of angiogenesis [5]. In the present study, we wanted to evaluate the effect of secreted HIF-1α from the ischemic vessels on angiogenesis pattern of the adjacent vessels which are non ischemic. To study the same, we followed the angiogenesis pattern of the vessels, which is just adjacent to the ischemic vessels. The images of the vessels at different time interval were acquired during the period of ischemia using a Nikon Cool Pix camera adapted with a stereo microscope. Images were then analyzed using Adobe Photoshop version 7.0 and Angioquant software [56].

### Morphology counting of the cells under ischemia

EAhy926 and H9C2 cells were incubated on the top of the ischemic vascular bed. After 2 h incubation, cells were imaged with Nikon Cool Pix camera adapted to Motic inverted microscope. For reversible experiment, cells were first imaged and incubated under ischemia. After treatment, cells were again imaged and kept on the top of a normal vascular bed for another 2 h. After 2 h incubation, cells were imaged. For RL, NAC and TRZ based recovery experiments, cells were pretreated with RL, NAC and TRZ for 15 min placed on the top of ischemic vessels treated with RL, NAC and TRZ respectively.

### RNA isolation and quantitative RT-PCR

Total RNA was isolated from ischemia treated EC and ischemic tissue itself using spin prep kit (Medox Inc) and quantified and normalized according to the band intensities. cDNA synthesis was performed on 200 ng of RNA using mulv reverse transcriptase (New-England Biolabs) and PCR was performed using 50 ng cDNA. The reactions were carried out for 30 cycles at 92°C denaturation for 1 min, 57°C annealing for 2 min and 72°C elongation for 3 min. The forward primer sequence is 5-AGATCTCGGCGAAGTAAAGAGTCTGAAGT-3 and the reverse primer sequence is 5-AGCATCCTGTACTGTCCTGTGGTGACTTGT-3 for human HIF-1α while forward primer sequence is 5- ACTTCAGCAGACTCAGAC-3 and the reverse primer sequence is 5-GTTCCAATGTTCCTTTTC-3 for avian HIF-1α, which amplifies the human and avian HIF-1α sequence (3376 bp–3691 bp) with a 209 bp product. The density of each band in the 2% agarose gel was quantified using Bio-RAD Gel Quantification software.

### Histology of the ischemia treated cardiac tissue samples

The biopsies were performed in the laboratory condition following the institutional and national ethical guideline. Minimum four endomyocardial samples were collected from different regions of the left side of the interventricular septum. Samples were then treated under ischemia by incubating it on ischemic vascular bed. For RL based recovery experiment, the heart tissues were pre-treated with RL for 15 min followed by incubation on ischemic vascular bed treated with RL during the period of incubation. After treatment, samples were fixed with 10% formaldehyde. Next, the samples of the non-ischemic and ischemic treated group were embedded in paraffin wax, sectioned into 5 µm slices and stained with the hematoxin-eosin stain. Next, the slides were observed under the 4X objective of a stereo microscope and imaged with Nikon Cool Pix camera adapted with the microscope. The technician responsible for examining the slides had no information about the sample types thus avoiding the biasness.

### Studying CK-MB activity of the ischemia treated heart tissue samples

Samples isolated using the above protocol was treated under ischemia. Next, the cardiac tissues were homogenized with 1X HBS (0.4 gm NaCl, 0.0135 gm Na_2_HPO_4._ 2H_2_O, 0.6 gm HEPES for 100 ml, pH-7.2) containing leupeptin (1 µM), aprotinin (1 µM), phenyl methane sulphonyl fluoride (1 mM). Briefly, samples were homogenized with HBS using the glass homogenizer. Next, the homogenized samples were centrifuged in 8000 g for 10 min. During the process of preparing samples the temperature was maintained at 4°C. Supernatants were taken out and stored in −85°C. CK-MB activities of the samples were measured with in 24 h of sample preparation. CK-MB activity was measured using the enzyme activity kit.

### Statistical analysis

All the experiments were performed in triplicate (n = 3) unless otherwise specified. Data is presented as mean + SE. Data was analyzed using the one-way ANOVA test, the Student *t*-test and the Tukey post hoc test, as appropriate. P values smaller or equal to 0.05 were used as the criterion for a statistically significant difference.
